# A novel mutation in *CFAP47* causes male infertility due to multiple morphological abnormalities of the sperm flagella

**DOI:** 10.3389/fendo.2023.1155639

**Published:** 2023-06-23

**Authors:** Mohan Liu, Siyu Dai, Jiying Zhang, Yihong Yang, Ying Shen, Hongqian Liu, Yanting Yang, Chuan Jiang, Erpo Tian

**Affiliations:** ^1^ State Key Laboratory of Biotherapy and Cancer Center, West China Hospital, Sichuan University and Collaborative Innovation Center, Chengdu, China; ^2^ Department of Obstetrics and Gynecology, West China Second University Hospital, Sichuan University, Chengdu, China; ^3^ Medical Genetics Department/Prenatal Diagnostic Center, West China Second University Hospital, Sichuan University, Chengdu, China; ^4^ Key Laboratory of Obstetric, Gynecologic and Pediatric Diseases and Birth Defects of Ministry of Education, Reproduction Medical Center of West China Second University Hospital, Sichuan University, Chengdu, China; ^5^ Department of Obstetrics/Gynecology, Key Laboratory of Obstetric, Gynecologic and Pediatric Diseases and Birth Defects of Ministry of Education, West China Second University Hospital, Sichuan University, Chengdu, China; ^6^ Department of Andrology, Xi’nan Gynecology Hospital, Chengdu, China

**Keywords:** asthenoteratozoospermia, MMAF, CFAP47, hemizygous mutation, WES

## Abstract

**Introduction:**

A previous study suggested that loss of CFAP47 function is involved in multiple morphological abnormalities of the sperm flagella (MMAF) in humans and mice. However, the comprehensive role of *CFAP47* in spermatogenesis is largely unknown.

**Methods:**

Whole-exome sequencing (WES) was conducted to identify pathogenic variant in two patients with MMAF. The functional effect of the identified mutations was investigated by immunofluorescence staining and western blotting. Intracytoplasmic sperm injection (ICSI) was used to assist fertilization for the patient with MMAF.

**Results:**

In this study, we identified a novel missense mutation (c.1414G>A; p.V472M) in *CFAP4*7 in two unrelated patients with oligoasthenoteratozoospermia. Intriguingly, in addition to the MMAF phenotype very analogous to the previous report, the two patients notably presented abnormal morphology of sperm heads, the sperm mitochondrial sheath was obviously disorganized, and the sperm annulus were almost defective. Further functional experiments confirmed that the expression of CFAP47 was markedly reduced in the spermatozoa of the patients. Mechanism analysis suggested that CFAP47 might regulate the expression of CFAP65, CFAP69 and SEPTIN4 through their physical interactions and thus modulating sperm morphogenesis.

**Conclusion:**

we revealed a novel mutation in *CFAP47* and further expanded the phenotype and mutation spectrum of *CFAP47*, as well as the potential mechanism of *CFAP47* manipulating spermatogenesis, finally providing important guidance for genetic counseling and targeted treatment for *CFAP47* mutation-related male infertility.

## Introduction

Infertility refers to a couple failing to conceive after 12 months of unprotected regular sexual intercourse (1, 2). It affects approximately 15% of couples worldwide, and male factors contribute to more than half of the cases (3). Spermatogenesis disorders include sperm defects in quality or quantity, which is manifested as the decreased sperm number, reduced sperm motility or abnormal sperm morphology (4). According to the criteria published in 2010 by the World Health Organization (WHO), teratozoospermia is defined as the presence of spermatozoa with abnormal morphology over 96% in one-time ejaculation (5). The phenotype and etiology of teratozoospermia are highly heterogeneous, and recent research based on animal models and genetic analysis has uncovered certain disease-causing or disease-promoting genes (6). Sperm head abnormalities are observed in teratozoospermia patients, mainly including macrozoospermia and globozoospermia. *Aurora kinase C* (*AURKC*) is the only definite cause of macrozoospermia (7); dysfunction of *dpy-19 like 2* (*DPY19L2*) (8), *chromosome 7 open reading frame 61* (*C7orf61*) (9), *c2 calcium-dependent domain containing 6* (*C2CD6*) (9), *gametogenetin* (*GGN*) (9), *coiled-coil domain containing 62* (*CCDC62*) (10), *calicin* (*CCIN*) (11), *protein interacting with PRKCA 1* (*PICK1*) (12), *spermatogenesis associated 16* (*SPATA16*) (13) and *zona pellucida binding protein 1* (*ZPBP1*) (14) are associated with globozoospermia. Multiple morphological abnormalities of the sperm flagella (MMAF) is a kind of asthenoteratozoospermia with defects in the sperm tail and reduced sperm motility (progressive motility of spermatozoa less than 32%) (5). Thus far, 42 MMAF-associated genes have been identified (6, 15, 16). However, a large number of asthenoteratozoospermia cases could not be explained. Therefore, a more comprehensive investigation of the pathology and molecular mechanisms of asthenoteratozoospermia is needed to further boost diagnosis efficacy.

Recently, Liu et al. suggested that *Cilia and flagella-associated protein 47* (*CFAP47*) is an MMAF-associated gene (17). They showed that patients with hemizygous *CFAP47* variants exhibit a typical MMAF phenotype, and the sperm ultrastructure shows an abnormal axoneme, including disorganized outer dense fibers (ODF), peripheral microtubule doublets (DMTs), and central pair of microtubules (CPs) (17). *CFAP47*-mutated male mice are sterile, with decreased sperm motility and abnormal flagella morphology (17). Therefore, the involvement of *CFAP47* in spermatogenesis is proved, and future studies should evaluate *CFAP47* mutations in larger cohorts to explore the comprehensive function of *CFAP47* in sperm morphology development.

Herein, we reported two infertile patients who carried a novel hemizygous mutation c.1414G>A [p.V472M] in *CFAP47*. Bioinformatics analysis and functional studies *in vitro* supported the pathogenicity of this mutation. Intriguingly, aside from the typical MMAF phenotype, numerous malformed sperm heads, defective sperm annulus, and aplasia sperm mitochondrial sheaths were evidently observed in the patients by exhaustive morphology analysis. Functional experiments indicated that CFAP47 might regulate the expression of CFAP65, CFAP69 and SEPTIN4 to mediate sperm morphogenesis. Our work highlighted a potential role of *CFAP47* in sperm head and tail formation in humans, broadening the gene variant and phenotype spectrum of *CFAP47* in male infertility.

## Materials and methods

### Study subjects and sample collection

The two infertile patients and their parents were recruited from the West China Second University Hospital of Sichuan University, and the chromosomal karyotypes of the patients were normal. This study was conducted following the tenets of the Declaration of Helsinki, and ethical approval was obtained from the Ethical Review Board of West China Second University Hospital, Sichuan University. We obtained written informed consent from each study participant.

### Genetic analysis

Peripheral whole blood samples were collected from patients and their parents for genetic analyses. Using a whole blood DNA-purification kit (51104, QIAGEN), genomic DNA was extracted from peripheral blood samples of patients and their parents. Whole-exome sequencing (WES) and bioinformatics analyses were performed on the patients’ samples. One microgram of genomic DNA was used for exon capture utilizing the Agilent Sure Select Human All Exon V6 Kit (Agilent Technologies) and then sequenced on the Illumina HiSeq X system (Illumina). PCR amplification was accomplished with Dyad Polymerase (Bio-Rad Laboratories), and an ABI377A DNA sequencer (Applied Biosystems) was utilized to sequence the PCR products. Functional annotation was performed using ANNOVAR through a series of databases, including the 1000 Genomes Project, dbSNP, HGMD, and ExAC. Next, PolyPhen‐2, SIFT, and M-CAP were used for functional prediction. Subsequently, variants were ignored if (1) the minor allele frequency was ≥1% in the public database including the gnomAD, ExAC Browser, and 1000 Genomes Project because the pathogenic variants account for male infertility are rare in human populations; (2) the variant located in 3′ or 5′ untranslated regions, noncoding exons, or intronic sequences except splice sites; and (3) the variant was not predicted damaging by PolyPhen‐2, SIFT, and M-CAP. The primers used in PCR analysis were as follows: F, 5’-ACCATTATGAGCTAGCTTTCCTT-3’; and R, 5’-ACAGTAACAACAAAGCCAGGT-3’.

### Immunofluorescence staining

The sperm from patients and the normal controls and mouse sperm cells were fixed in 4% paraformaldehyde, permeabilized with 0.3% Triton X-100 for 10 min, and blocked with 5% BSA or 30% donkey serum for 60 min at room temperature. The slides were then sequentially incubated with primary antibodies at 4°C overnight. The primary antibodies used were anti-CFAP47 (1:50, sc-514714, Santa Cruz Biotechnology) and α-tubulin (1:100, A11126, Thermo Fisher Scientific), CFAP65 (1:50, HPA055156, Sigma–Aldrich), CFAP69 (1:50, bs-15278R-A647, Bioss), COXIV (1:50, 11242-1-AP, Proteintech) and SEPTIN4 (1:50, 12476-1-AP, Proteintech). The next day, 1 × PBS was used to wash the samples three times. Then, the samples were incubated with AlexaFluor 594 anti-rabbit secondary antibodies (1:1000, 1927937, Thermo Fisher) and AlexaFluor 488 anti-mouse secondary antibodies (1:1000; A32723, Thermo Fisher) for 2 h in room temperature or coincubated with peanut agglutinin (PNA, 1:50, RL‐1072‐5, Vector). Subsequently, we used 1 × PBS to wash the slides three times. Then, the slides were counterstained with 4,6-diamidino-2-phenylindole (DAPI, D9542‐1MG, Sigma‐Aldrich) to label the nuclei. Finally, the slides were sealed in coverslips. Images were obtained by a laser scanning confocal microscope (Olympus). For immunofluorescence staining of mouse testis, after careful xylene dewaxing and gradient ethanol rehydration, the tissue sections were submerged in boiling 10 mM citrate buffer (pH 6.0) for 10 min. Then, the sections were cooled to room temperature and washed with 1 × PBS for 5 min. Subsequently, the sections were treated with 3% hydrogen peroxide solution for 10 min. After washing with 1× PBS, the slides were blocked with 10% normal donkey serum for 30 min and incubated with primary antibodies at 4°C overnight and with Alexa Fluor 488 or Alexa Fluor 594 antibodies for an additional 2 h at room temperature. The primary antibodies used were anti-CFAP47 (1:50), anti-CFAP65 (1:50) and anti-CFAP69 (1:50). Slides of testicular tissues were observed using an LSM800 confocal microscope (Carl Zeiss AG).

### Western blotting

Proteins were isolated from sperm cells. Protein quantitation was performed by a BCA Protein Assay (23227, Thermo Fisher) according to the manufacturer’s instructions. Next, protein denaturation was performed at 100°C for 10 min. The denatured proteins were separated on 10% SDS-polyacrylamide gels. Then, these proteins were transferred into a 0.45 µm pore size polyvinylidene difluoride (PVDF) membrane (ISEQ, 00010, Millipore) by wet transfer. Subsequently, 5% skimmed milk was used to block the transferred membrane. Next, the transferred membrane was incubated in primary antibody: anti-CFAP47 (1:500), anti-CFAP65 (1:1000) and anti-CFAP69 (1:500), anti-GAPDH (1:1000, ab8245, Abcam) solution at 4°C overnight. Subsequently, the membrane was washed with 1 × TBST three times. Then, the membrane was incubated with goat anti-mouse IgG secondary antibody-HRP (1:5000, 32230, Thermo Fisher Scientific) in 5% skimmed milk at room temperature for 1 h and then wash the membrane with 1 × TBST three times. Finally, immunoblots were developed using Thermo Scientific™ Pierce™ ECL Western Blotting Substrate (TWBKLS0500, Millipore).

### Electron microscopy and concentrated Papanicolaou staining

For scanning electron microscopy (SEM), the sperm samples were centrifuged at 400×g for 10 min at room temperature. The supernatants were carefully aspirated, and the pellets were suspended and fixed in 2.5% glutaraldehyde for 30 min at 4°C. Next, the samples were evenly spread onto slides and fixed in 2.5% glutaraldehyde overnight at 4°C. Following primary fixation, the slides were washed three times in 1×PBS and gradient dehydration was performed sequentially with 30%, 50%, 75%, 95%, and 100% ethanol for 10 min. Subsequently, the slides were dried to temperature with a CO2 critical-point dryer (Eiko HCP-2, Hitachi). Finally, all of the dried specimens were mounted on aluminum stubs, sputter-coated by an ionic sprayer meter (Eiko E-1020, Hitachi), and analyzed by SEM (Hitachi S3400).

For transmission electron microscopy (TEM), sperm samples were washed routinely and centrifuged at 400 × g for 15 min. Then, the seminal plasma was removed, and the sperm pellets were fixed in 3% glutaraldehyde. Next, the samples were postfixed in 1% buffered OsO4, dehydrated through gradient acetone solutions, and embedded in Epon 812. Finally, the ultrathin sections (80 nm) were double-stained with lead citrate and uranyl acetate before being observed and photographed via TEM (TECNAI G2 F20, Philips).

### Isolation of mouse spermatogenic cells

Spermatogenic cells were obtained through cell diameter/density at unit gravity. In brief, mouse germ cells were isolated from the testicular biopsy tissues of obstructive azoospermia patients with informed consents and 8-week C57BL/6 male mice, respectively. In order to remove cell aggregates, spermatogenic cells were resuspended in 25 ml of 0.5% BSA solution and filtered through an 80 mm mesh. The cells were resuspended in buffer containing 0.5% BSA and loaded in an STA-PUT velocity sedimentation cell separator (ProScience) for gradient separation after passage through a mesh filter. Germ cell populations were collected for subsequent analysis.

### Protein–protein interaction network

Standardize gene names from Uniprot Knowledgebase (UniprotKB, http://www.uniprot.org), selecting “Homo sapiens”. Built on the co-targets result, the PPI network was conducted by Search Tool for the Retrieval of Interacting Genes (STRING, http://cn.string-db.org/). We put the known MMAF causative genes and *CFAP47* into the STRING analysis. The relevant parameter settings are as follows: 1) Network Type: full STRING network; 2) Required score: highest confidence (0.900); 3) Size cutoff: no more than 10 interactors.

### Co-immunoprecipitation

The protein collected from human testes from obstructive azoospermia patients with informed consents was incubated with 7 μl of target antibodies overnight at 4°C. Subsequently, we added the mixture of each sample to a microcentrifuge tube containing 40 μL of prewashed Protein A/G magnetic beads (88803, Invitrogen), and the samples were incubated for 2 h at room temperature with constant rotation. After washing with 1 × PBS three times, the coimmunoprecipitated proteins were eluted with standard 5 × SDS sample loading buffer and heated for 10 min at 100°C. Finally, the co-immunoprecipitants were separated on 10% SDS-polyacrylamide gels and PVDF membranes for immunoblot analysis, as described above.

## Results

### Identification of a *CFAP47* missense variant in two unrelated infertile men

**Figure 1:**
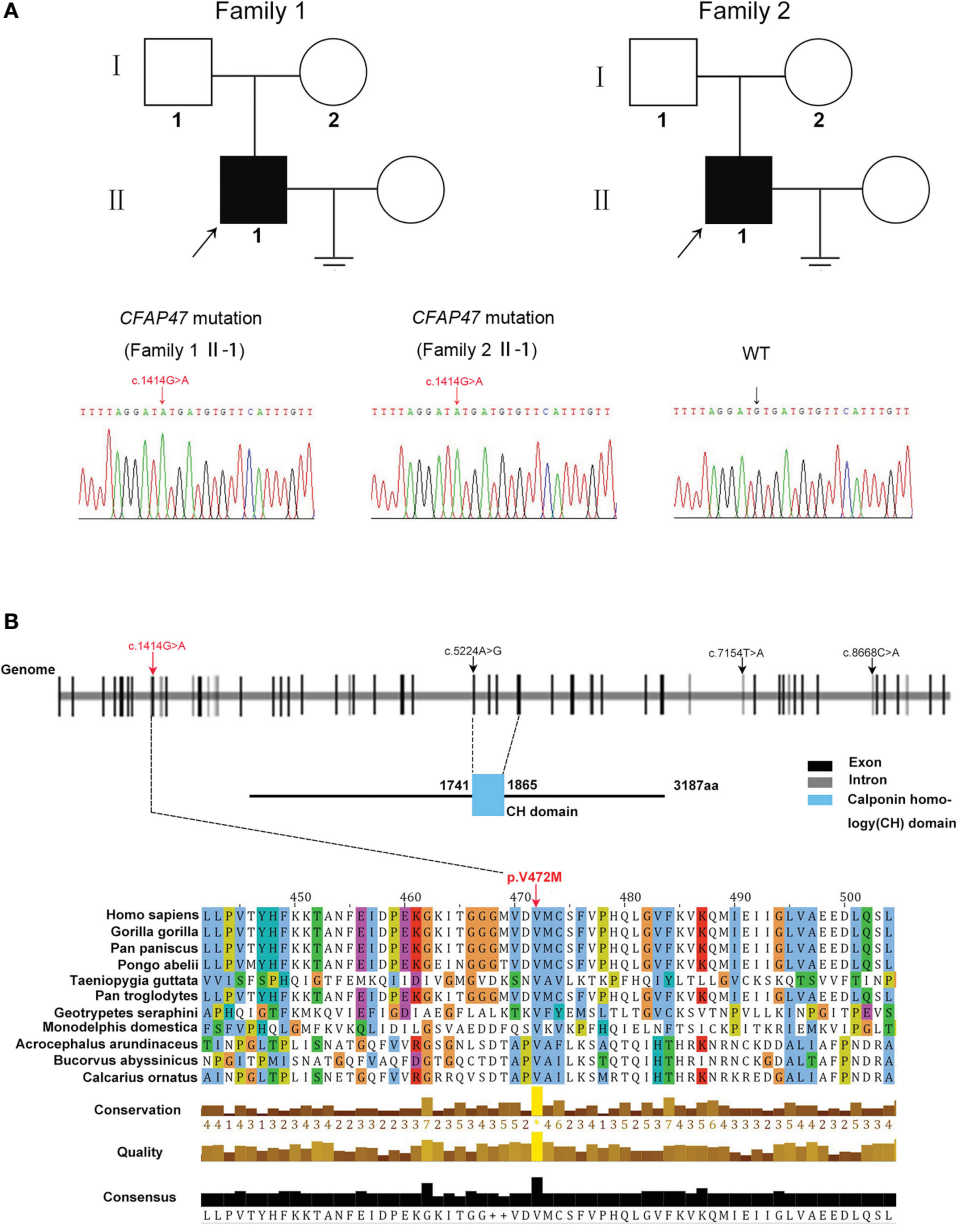
Identification of a novel variant of X-linked *CFAP47* in oligoasthenoteratozoospermia patients. **(A)** Pedigree structure of two families affected oligoasthenoteratozoospermia. The probands are indicated by black arrows. Sanger sequencing confirmed a hemizygous *CFAP47* missense variant in the two families. The detailed position of the variant (c.1414G>A) is indicated by red arrows. **(B)** CFAP47 protein structure, localization of variants in the genome, and conservation of mutant amino acids in various species. The red arrow indicates the position of the mutation in our study. The black arrow indicates the mutations that have been reported. Residue V472 is conserved across species.

Two oligoasthenoteratozoospermia patients were recruited for our study. Whole-exome sequencing was next performed on the two patients (**Figure 1A**). Intriguingly, a novel missense mutation of c.1414G>A [p.V472M] in *CFAP47* was identified in both patients (**Figure 1A**). This *CFAP47* mutation has a low allele frequency in East Asian populations in public databases (**Table 1**) and was predicted to be harmful through the prediction of SIFT, PolyPhen-2, and M-CAP tools (**Table 1**). Subsequent sequence alignment analysis found that this amino acid was conserved in multiple species (**Figure 1B**). These findings indicated that this hemizygous mutation in *CFAP47* might be a potential pathogenic factor for the sterile phenotype of patients.

**Table 1:** Semen and variant analysis in the two patients harboring hemizygous a *CFAP47* mutation.

		P1	P2	References
	Sperm volume (mL)	1.9	3.8	>=1.5
	Sperm concentration(10^6^/mL)	6.0	0.9	>=15
	Motility (A+B, %)	21	11.0	>=40
**Semen**	Vitality (%)	1.0	5.8	>=58
**parameters**	Normal spermatozoa (%)	1	2.7	>=4
	Defective spermatozoa (%)	99	97.3	
	pyriform head (%)	18	37	
	Round head (%)	32	29	
	Coiled flagella (%)	39	47	
	Absent flagella (%)	5	9.4	
	Bent flagella (%)	52	34.9	
	cDNA mutation	c.1414G>A	c.1414G>A	
	Protein changes	p.V472M	p.V472M	
	Mutation type	Missense	Missense	
	Genotype	Hemizygous	Hemizygous	
**Variants**	Allele frequency			
**analysis**	in ExAC Browser (ExAC_EAS)	0.0091	0.0091	
	GnomAD (gnomAD_exome_EAS)	0.0094	0.0094	
	1000 Genomes Project (1000g2015aug_eas)	0.0092	0.0092	
	Function Prediction			
	SIFT	Deleterious	Deleterious	
	Polyphen-2	Probably damaging	Probably damaging	
	M-CAP	Deleterious	Deleterious	

RefSeq accession number of CFAP47: NM_001304548.

### Presentation of MMAF in two infertile patients

**Figure 2:**
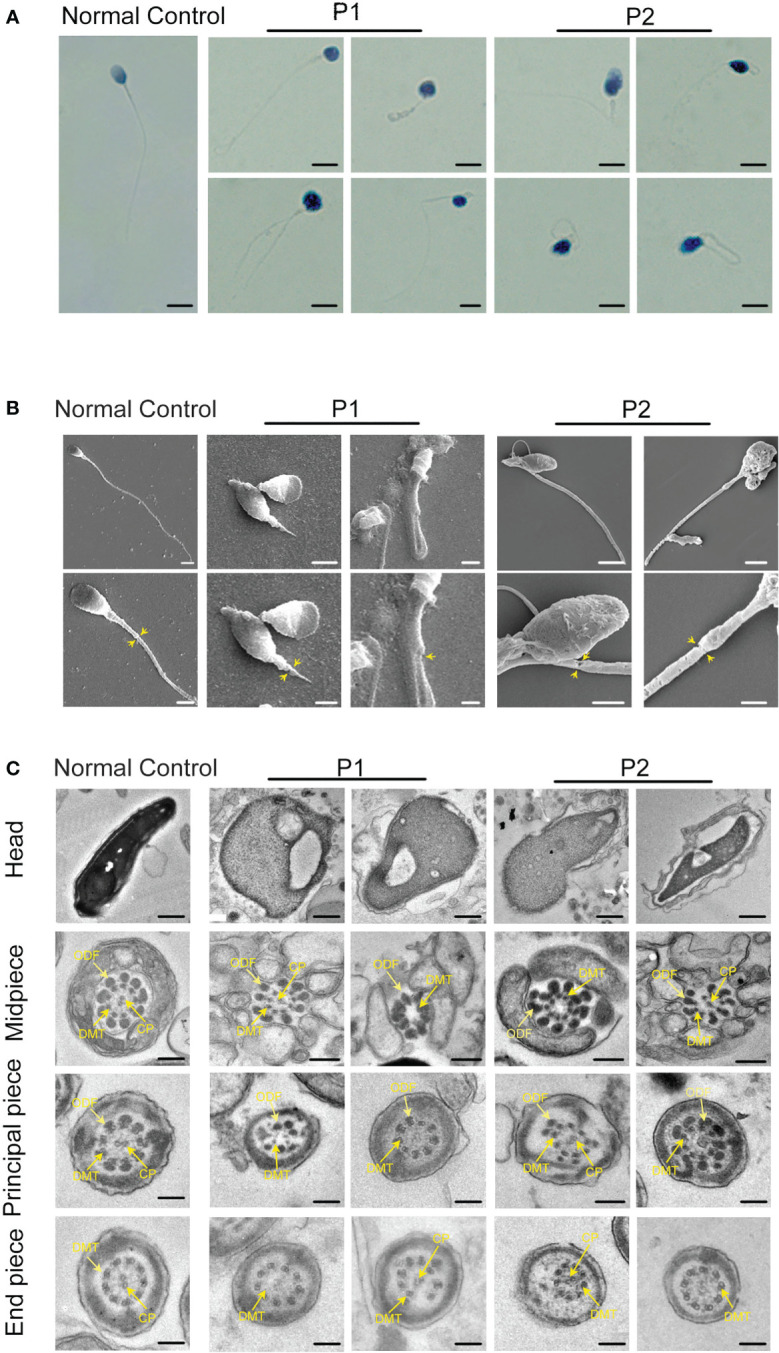
Sperm morphology and ultrastructure analysis for patients harboring hemizygous *CFAP47* variant. **(A)** Papanicolaou staining of spermatozoa obtained from normal control and patients. The patients’ sperm showed irregular morphology (Scale bars, 5 µm). **(B)** Morphological defects in sperm were observed in the patient by SEM. The abnormal annulus of patients is indicated by yellow arrows (Scale bars, 5 µm). **(C)** TEM showed abnormal ultrastructure of the head and flagellum from the patients’ spermatozoa compared to normal control (ODF, outer dense fibers; DMT, peripheral microtubule doublet; CP, central pair of microtubules; Scale bars, 200 nm).

To explore the detailed infertile phenotypes in the two patients, we performed semen analysis according to WHO guidelines. The results indicated dramatic decreases in sperm count, sperm motility accompanied by aberrant sperm morphology (**Table 1**). Papanicolaou staining and SEM were further used to analyze sperm morphology in detail. Unlike the normal control, the spermatozoa from the patients exhibited a typical MMAF phenotype, including absent, short, and coiled flagella (**Figure 2A, B**). Strikingly, the patients’ sperm also exhibited anomalous morphology in the sperm head, such as pyriform head, small head, or round head (**Figure 2A, B**). Noticeably, the impaired conjunction between the midpiece and principal piece was apparent, suggesting that the patients might have defective sperm annulus (**Figure 2B**).

**Figure 3:**
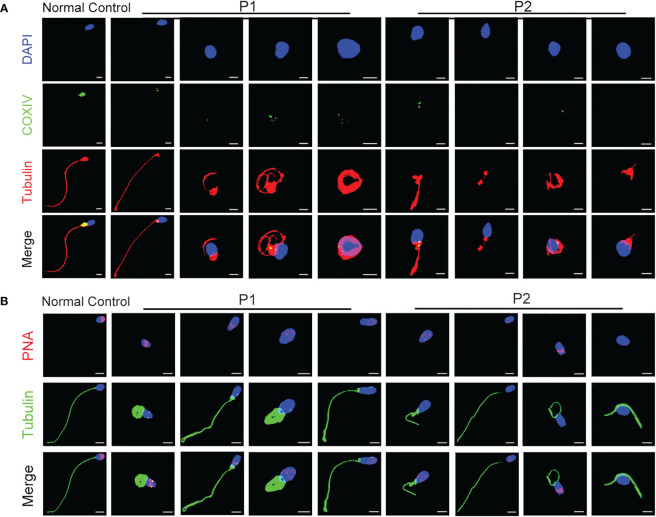
The characterization of spermatozoa in patients. **(A)** COXIV immunostaining disappeared in the sperm of patients compared to the normal control (Blue, DAPI; green, COXIV; red, α-tubulin; scale bars, 5 μm). **(B)** The immunostaining of PNA exhibited imperfect acrosomes in sperm cells of patients compared to the control subject (Blue, DAPI; green, α-tubulin; red, PNA; scale bars, 5 μm).

TEM was performed to investigate the ultrastructure of spermatozoa. In contrast with the well-organized peri-axonemal and axonemal structures in the normal sperm flagella, the sperm flagella of the patients showed severe disorganization or absence of the peri-axonemal and ‘9 + 2’ axonemal components and the disarrangement of mitochondrial sheaths and dense fibers (**Figure 2C**). Strikingly, typically swollen mitochondria were present in the middle piece (**Figure 2C**). Based on this phenomenon, COXIV, a marker of mitochondrial sheath integrity, was used to analyze defects in the mitochondrial sheath. In control sperm, COXIV localized to the midpiece of sperm flagella, but it disappeared completely in the sperm of patients (**Figure 3A**). Intriguingly, most patients’ spermatozoa exhibited irregular and unconsolidated nuclei (**Figure 2C**). Additionally, the majority of the acrosomes were small or even absent in the sperm heads of the patients (**Figure 2C**). Immunofluorescence staining of PNA also demonstrated disrupted or absent acrosomes in the sperm of patients (**Figure 3B**). All evidence indicates that *CFAP47* mutation contributes not only to the MMAF phenotype but also to abnormalities in the sperm head and annulus.

### The deleterious effect of this missense variant on CFAP47 expression and function

**Figure 4:**
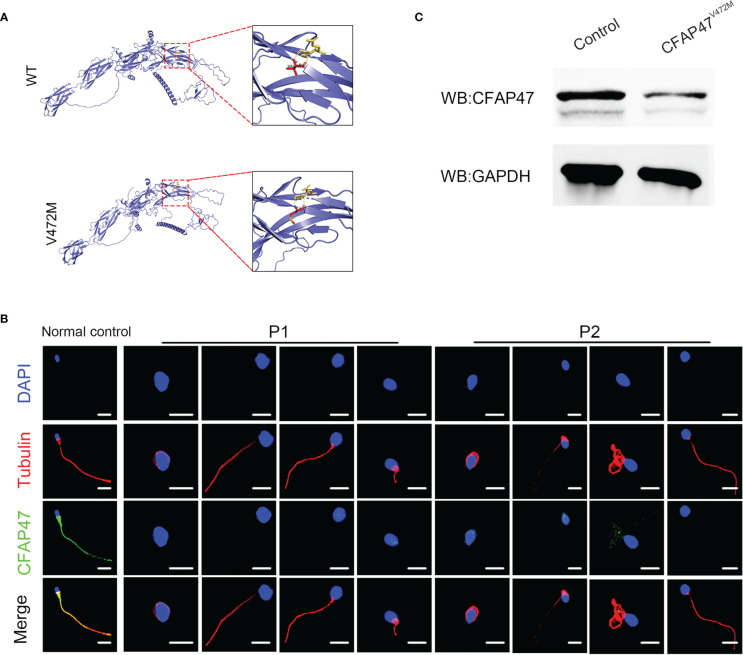
Expression analysis of *CFAP47* in the spermatozoa from a male control individual and men harboring hemizygous *CFAP47* variants. **(A)** Structural illustration of the missense mutation in CFAP47. **(B)** Immunoblotting assays revealed that CFAP47 was dramatically reduced in the spermatozoa of patients harboring *CFAP47* mutation. **(C)** Immunofluorescence staining reflected a marked decline in CFAP47 expression in the patients’ sperm compared with that in the normal control (Blue, DAPI; red, α-tubulin; green, CFAP47; scale bars, 5 μm).

To investigate the impact of this variant on CFAP47 protein structure, PyMOL Viewer software was used to visualize the effects of altered residues on protein-structure models (**Figure 4A**). The amino acid sequence of predicted structure of CFAP47 included the residues from 1 aa to 754 aa. Mutant V472M showed it may affect the stability of the original β-sheet region for methionine occupied more space than valine, indicating that the structure of CFAP47 was disordered. To further determine the impact of c.1414G>A on CFAP47 expression, we detected CFAP47 expression in the patients’ spermatozoa via immunofluorescence staining. CFAP47 expression was mainly distributed in flagella in control spermatozoa, while CFAP47 staining was barely detected in the spermatozoa of patients (**Figure 4B**). Meanwhile, western blotting showed similar results of significantly decreased protein expression of CFAP47 in the patients’ sperm lysates compared to the fertile control (**Figure 4C**).

**Figure 5:**
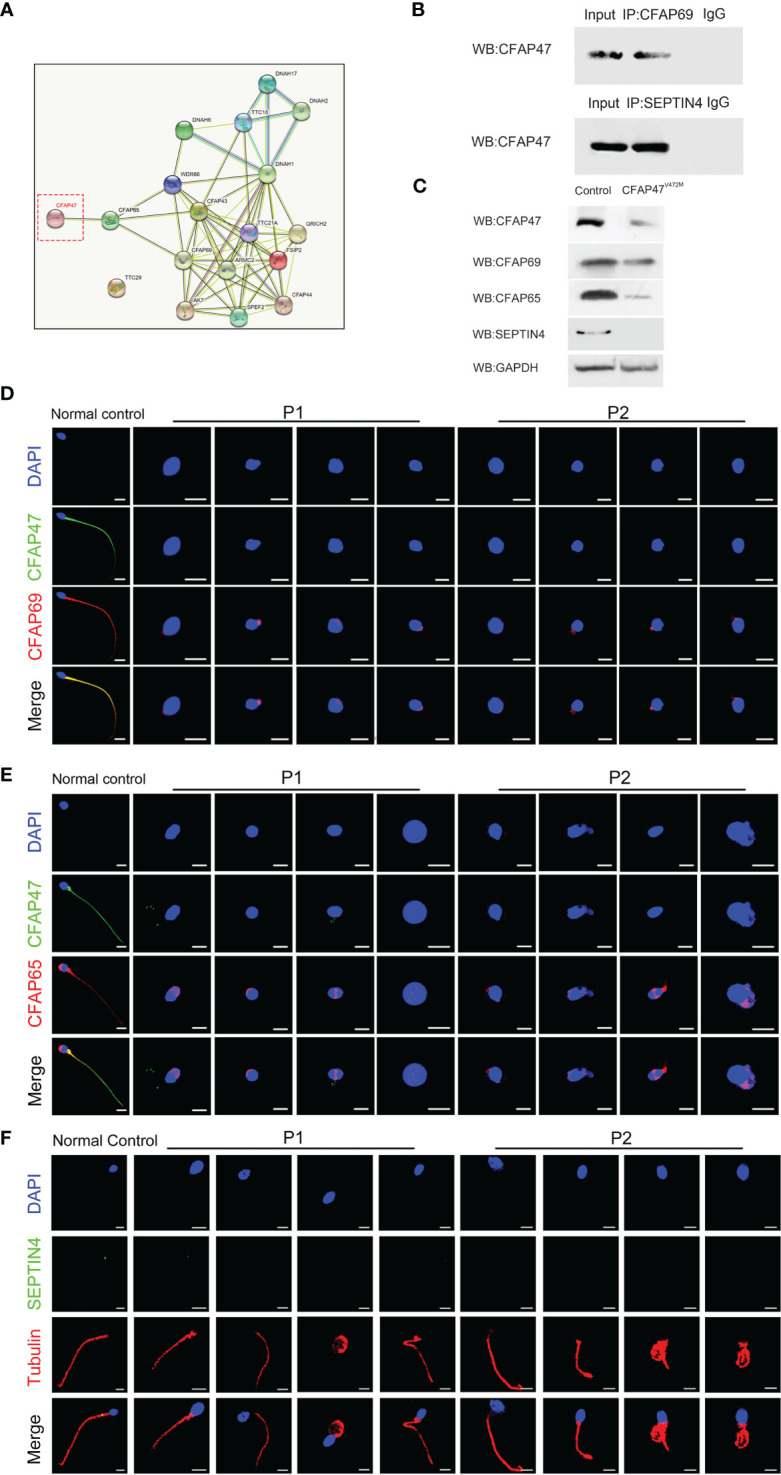
The altered expression of key molecules involved in spermatogenesis is mediated by CFAP47. **(A)** The protein interaction network was predicted by in silico software STRING for the human CFAP47 protein. Known interactions: the light blue lines symbolize that the two connected proteins are from curated databases, and the red lines represent that the proteins are experimentally determined; Predicted interactions: the green lines symbolize that the two connected proteins are gene neighborhood, the orange lines represent that the proteins are gene fusions, and the dark blue lines represent that the proteins are gene co-occurrence; Others: the yellow lines symbolize that the two connected proteins are textmining, the black lines represent the co-expression proteins, and the gray lines represent the protein homology. **(B)** Co-immunoprecipitation analysis showing the binding of CFAP47 with CFAP69 and SEPTIN4 using human testis lysates. **(C)** Immunoblotting assays revealed that CFAP65 and CFAP69 were dramatically reduced in the spermatozoa of patients harboring *CFAP47* mutations. **(D, E)** The signals of CFAP69 **(D)** and CFAP65 **(E)** were clearly reduced in the sterile patients by fluorescence detection (Blue, DAPI; green, CFAP47; red, CFAP65 and CFAP69; scale bars: 5 μm). **(F)** Expression of SEPTIN4 was not visible in the sperm annulus of patients compared to normal control (Blue, DAPI; green, SEPTIN4; red, α -tubulin; scale bars, 5 μm).

STRING analysis (https://cn.string-db.org/) revealed that CFAP47 may be connected with CFAP69, a key molecule involved in sperm flagellar formation (**Figure 5A**) (18). Co-immunoprecipitation further verified the binding between CFAP47 and CFAP69 in human testis lysates (**Figure 5B**). In addition, a previous study demonstrated that CFAP47 regulated and interacted with CFAP65 (17), which has been suggested to regulate sperm head development (19). Strikingly, the expression levels of CFAP69 and CFAP65 were validated to be sharply reduced in the sperm of the two patients compared to the normal control via immunofluorescence staining and western blotting (**Figure 5C-E**). In addition, the immunofluorescence assay confirmed the colocalization of CFAP47 with CFAP65 and CFAP69 in mouse spermatogenic cells at different stages (**Supplemental Figure 1, 2**) (20), as well as mouse testis sections (**Supplemental Figure 3**). We further detected the expression and localization of SEPTIN4, an essential protein for sperm annulus formation (21), by immunofluorescence staining in the patients’ sperm. As expected, the SEPTIN4 signal was located in the annulus in normal control but was almost disappeared in patients (**Figure 5F**). Co-immunoprecipitation further confirmed the physical interactions between CFAP47 and SEPTIN4 (**Figure 5B**), suggesting that CFAP47 might also be related to sperm annulus formation by regulating SEPTIN4. These results demonstrated that CFAP47 might regulate sperm morphology development by modulating the expression of CFAP65, CFAP69 and SEPTIN4.

### Outcomes of intracytoplasmic sperm injection in patients carrying *CFAP47* mutation

ICSI is a commonly used assisted reproductive technology (ART) to help sterile patients (22). ICSI cycles were attempted for our patients, and written informed consent was obtained for the procedure (**Table 2**). For patient A, his wife was followed up for one ICSI cycle—with eight oocytes retrieved after gonadotrophin-releasing hormone (GnRH) treatment. Five mature oocytes (metaphase II, MII) were collected, and one 8 II (the embryo has eight cells, the blastomere is uniform, and the fragment is 10% to 20%) was transferred. Regrettably, his wife has not succeeded in pregnancy. For patient B, his wife experienced a long GnRH agonist protocol in the first cycle. Six oocytes were retrieved, four mature oocytes were microinjected successfully, and only one oocyte was normally fertilized (1PN/injected oocytes = 25%). Following extended culture, we obtained one available D3 embryo that failed to develop after being transferred. After this progress, this couple continues the second cycle and chooses the antagonist protocol. We retrieved three metaphase II oocytes and injected them; however, they failed to develop after reaching the available D3 stage. However, a previous study reported satisfactory ICSI outcomes of a loss-of-function mutation in *CFAP47* in humans and male mice (17). We speculated that additional female risk factors for infertility should not be excluded, and more cases need to be investigated to clarify the role of this mutation in ICSI outcomes.

**Table 2:** Clinical features of the patients’ spouses with ICSI treatment.

		Spouse of P1	Spouse of P2
Age(y)		25	28
Length of primary infertility history (y)		3	4
BMI		18.4	19.1
Basal hormones	FSH (IU/L)	7.6	11.1
	LH (IU/L)	3.3	6.9
	E2 (pg/mL)	15.7	38.9
	PRL (ng/ml)	9.2	13.3
	Prog (ng/ml)	0.42	0.7
	Testo (ng/ml)	0.36	0.41
Cycle 1	Protocol	Long	Long
	E2 level on the trigger day (pg/mL)	2585	602
	No. of follicles ≥ 14 mm on the trigger day	5	2
	No. of follicles ≥18 mm on the trigger day	3	1
	No. of oocytes retrieved	8	6
ICSI progress	Oocytes injected	5	4
	Fertilization rate (%)	60% (3/5)	25% (1/4)
	Cleavage Rate (%)	100% (3/3)	100% (1/1)
	Available D3 embryos	1	1
Cycle 2	Protocol		Antagonist
	E2 level on the trigger day (pg/mL)		13.3
	No. of follicles ≥ 14 mm on the trigger day		5
	No. of follicles ≥18 mm on the trigger day		1
	No. of oocytes retrieved		3
ICSI progress	Oocytes injected		3
	Fertilization rate (%)		66.7% (2/3)
	Cleavage Rate (%)		100% (2/2)
	Available D3 embryos		1

## Discussion

In our study, we highlighted that in addition to MMAF, *CFAP47* mutation is associated with severe defects in other spermatozoa morphology, including sperm head, annulus and mitochondria. Furthermore, CFAP47 was first demonstrated to interact with CFAP69 and SEPTIN4, and further mediate their expression. With our experimental data, we suggest that *CFAP47* may be involved in sperm morphogenesis both in head development and flagellum assembly.

A previous study on an animal model reported a necessary role of *Cfap47* in spermatogenesis in mice, and their patients with *CFAP47* mutations were infertile, characterized by abnormal sperm motility and sperm flagellum morphology (17). However, the phenotype and mutation spectrum of *CFAP47* in humans have not been comprehensively studied. The underlying mechanism by which *CFAP47* regulates reproductive biology is also largely unknown. In the present study, we detected a novel missense mutation of *CFAP47* in two sterile patients from two unrelated families. By a comprehensive morphology analysis, we first suggested that *CFAP47* mutation is also linked to the abnormal sperm annulus and head morphology. Moreover, our mechanistic study found that CFAP47 could interact with CFAP65, CFAP69 and SEPTIN4 to further regulate their expression. In particular, CFAP69 and CFAP65, as known MMAF pathogenic genes, are required for sperm morphogenesis. Wang et al. reported that CFAP65 is expressed in the acrosome area and flagellar midpiece in normal human spermatozoa (19), and CFAP69 is localized to the sperm flagellum (18). SEPTIN 4 is essential for the structural and mechanical integrity of the spermatozoa annulus (21). Therefore, our findings demonstrated a potential mechanism by which CFAP47 might regulate spermatogenesis by mediating the expression of CFAP65, CFAP69 and SEPTIN4. In fact, there are a few genes that show phenotype heterogeneity to a certain extent. For example, *FSIP2* was initially reported as a pathogenic gene of MMAF (23), while Zheng et al. confirmed that deleterious mutations in *FSIP2* are responsible for abnormal acrosome biogenesis (24); loss-of-function mutations in *DNAH6* (25) were first detected in three MMAF patients by Tu et al. but also screened compound heterozygous variants in *DNAH6* in a patient who presented sperm head anomaly (26). These studies strongly suggested that some pivotal proteins may not perform a single function but also play a various role during spermatogenesis.

Sex chromosomes play a key role in sex determination and reproductive function (27). Other than genes located in the autosomal chromosome, sex chromosome genes lack corresponding alleles in males. Therefore, harmful mutations in sex chromosome genes have a direct impact on male infertility occurrence. To date, limited causative genes in sex chromosomes have been acknowledged. Partial deletion of *testis expressed 11 (TEX11)* leads to azoospermia due to meiotic arrest (28); mutations in *androgen receptor (AR)* attenuate AR regulation of target gene expression and cause oligozoospermia and azoospermia (29); dysfunction of *adhesion G protein-coupled receptor G2* (ADGRG2) results in a buildup of fluid within the testis and an accumulation of spermatozoa within the efferent ducts (30); *PIH1 domain containing 3* (*PIH1D3*) is involved in the assembly of the dynein arm of the ciliary axoneme, and defects in this gene lead to primary ciliary dyskinesia (PCD) (31); *Ubiquitin specific peptidase 9 Y-linked* (*USP9Y*) is expressed specifically in testis in a germ cell-dependent fashion and the absence of USP9Y has been suggested to cause spermatogenic failure (32). Hence, more sex chromosome gene pathogenicity and biological functions in male reproduction need to be explored. In the current study, we identified a novel homozygous mutation in *CFAP47*, which is located on the X chromosome, in two infertile patients. Our findings expanded the causative mutation of male infertility on the X chromosome, providing more valuable information for the diagnosis and treatment of male infertility.

In conclusion, our study identified a novel missense mutation in *CFAP47* in two infertile male patients with various sperm morphology abnormalities, first revealing *CFAP47* as a candidate causative gene of sperm multiple morphological abnormalities. Functional analysis demonstrated the underlying molecular mechanism by which CFAP47 regulates sperm morphogenesis. Our work presented more detailed information on the pathogenesis of *CFAP47* mutation and provided direct evidence that suggests the involvement of *CFAP47* in spermatogenesis.

## Data availability statement

The datasets presented in this article are not readily available because the datasets generated during and analyzed during the current study are available from the corresponding author on reasonable request. Requests to access the datasets should be directed to ET, tianep@jxr-fertility.com.

## Ethics statement

The studies involving human participants were reviewed and approved by Ethics Committee of the Second West China Hospital of Sichuan University. This study was performed in line with the principles of the Declaration of Helsinki. The patients/participants provided their written informed consent to participate in this study.

## Author contributions

ET supervised the study experiments. YiY conducted the clinical evaluations. YS analyzed the WES data. SD and JZ performed immunofluorescence staining. ML, YaY, HL, and CJ performed TEM and SEM. ML and SD provided figures and writing guidance. ML wrote the first article draft. ET revised the manuscript. All authors contributed to the article and approved the submitted version.
