# Supplementary material for: A novel mutation in CFAP47 causes male infertility due to multiple morphological abnormalities of the sperm flagella
Source: Front Endocrinol (Lausanne). 2023 Jun 23;14:1155639. doi: 10.3389/fendo.2023.1155639 (PMC10326514; doi:10.3389/fendo.2023.1155639)
Supplement: Supplementary file 1 [file DataSheet_1.docx]

**Supplemental Materials**


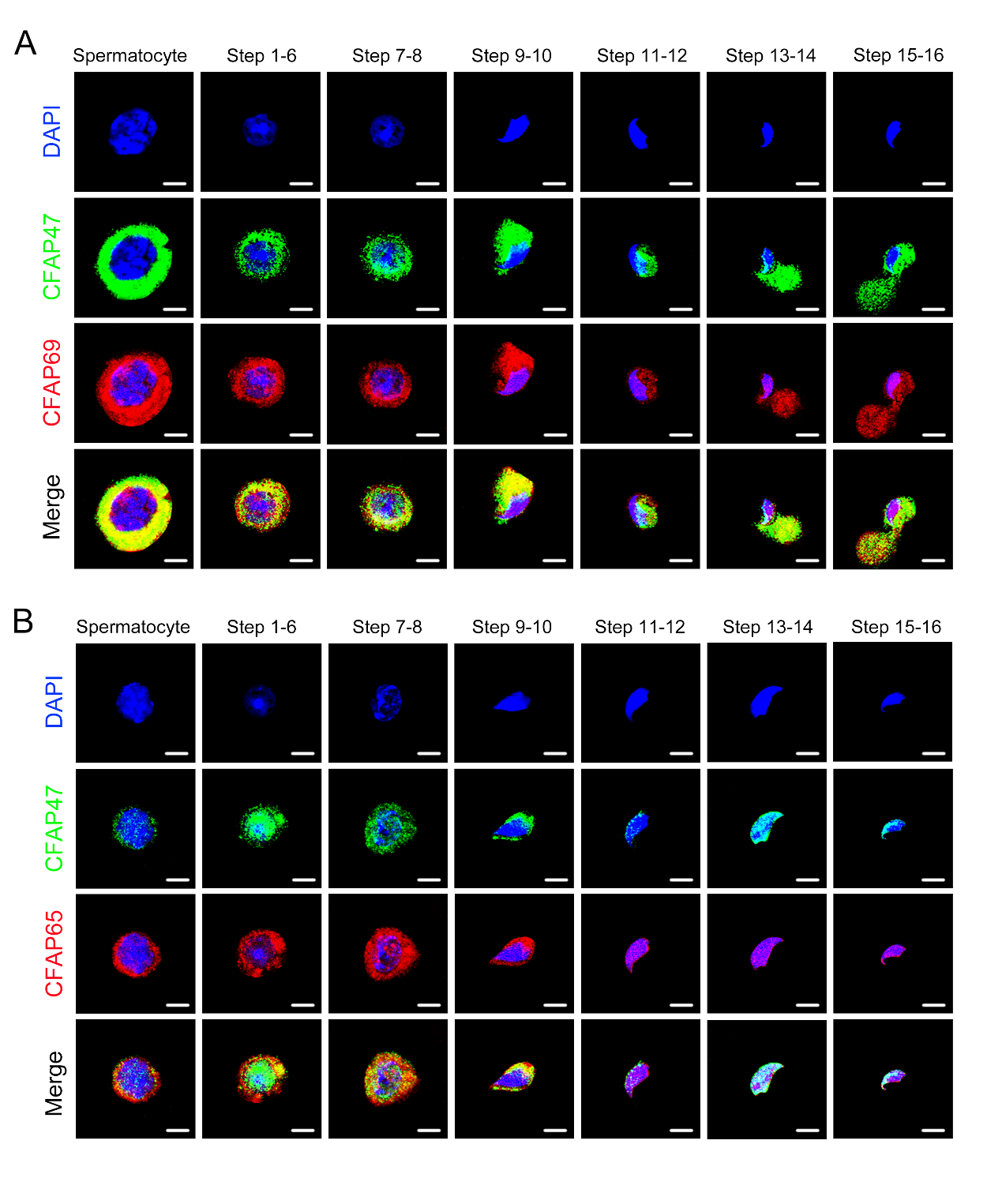
 **Supplemental Fig. S1** The colocalization of CFAP47 and CFAP69/CFAP65 in mouse germ cells. (A, B) Immunofluorescence images showing the colocalization of CFAP47 with CFAP69 (A) and CFAP65 (B) in various germ cells during mouse spermiogenesis (Blue, DAPI; green, CFAP47; red, CFAP65 and CFAP69; scale bars: 5 μm).


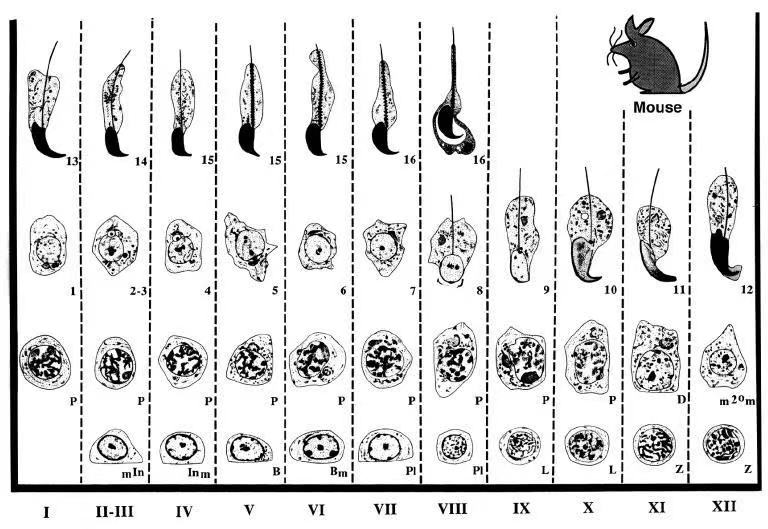


**Supplemental Fig. S2** Spermatogenic cycle of mouse. In mouse seminiferous tubules of the testis during spermatogenesis, the 12 cell stages that can be observed. Details of symbols used in the staging maps shown can be found in *Russell et al.* [20].


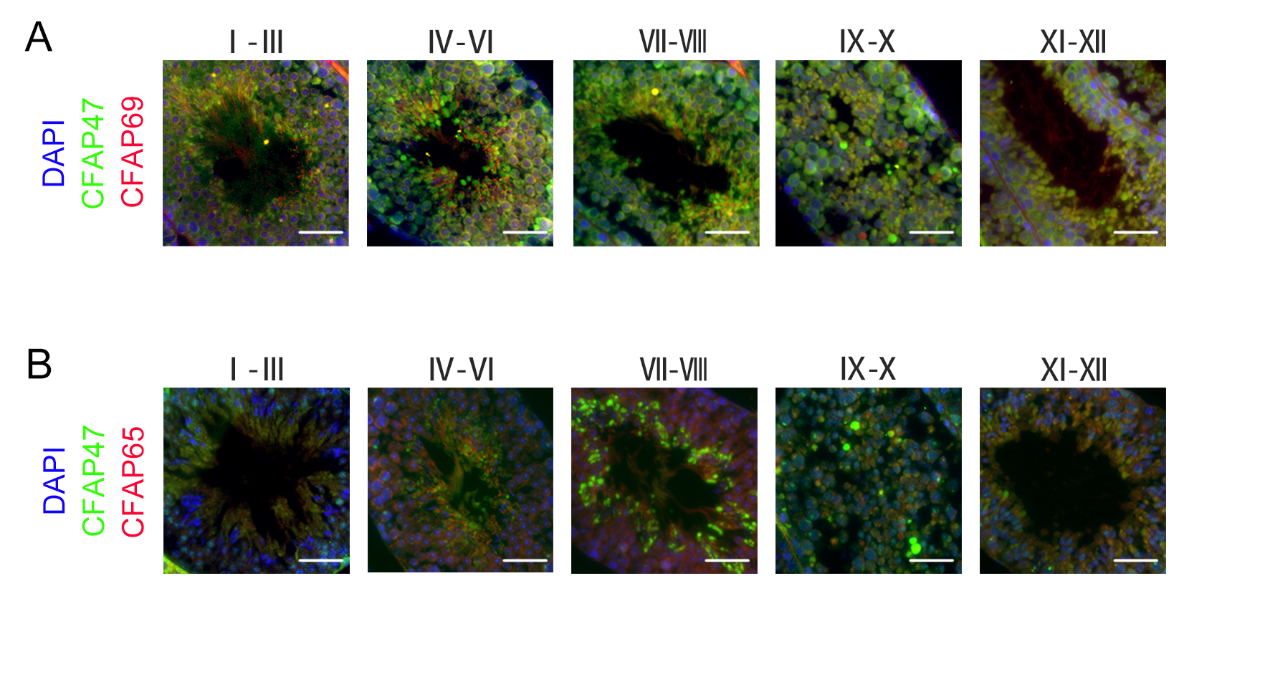
 **Supplemental Fig. S3** The colocalization of CFAP47 and CFAP69/CFAP65 in mouse testis. (A, B) Immunofluorescence images showing the colocalization of CFAP47 with CFAP69 (A) and CFAP65 (B) in the testes of mice at different stages (Blue, DAPI; green, CFAP47; red, CFAP65 and CFAP69; scale bars: 5μm
